# (2*R*,5*S*)-5-Benzyl-2,3-dimethyl-4-oxo-2-phenyl­imidazolidin-1-ium chloride

**DOI:** 10.1107/S1600536809010460

**Published:** 2009-03-28

**Authors:** Shuai Zhang, Yifeng Wang, Bailin Li, Guangcun Zhang, Shuping Luo

**Affiliations:** a State Key Laboratory Breeding Base of Green Chemistry–Synthesis Technology, Zhejiang University of Technology, Hangzhou 310014, People’s Republic of China; b Department of Pharmaceutical and Chemical Engineering, Taizhou College, Linhai, Zhejiang 317000, People’s Republic of China

## Abstract

The title hydro­chloride salt, C_18_H_21_N_2_O^+^·Cl^−^, is an imidazolidinone catalyst, which was derived from L-phenyl­alanine through cyclization with acetophenone. The imidazolidinone compound has a five-membered heterocyclic ring including two chiral centres. The imidazolidinone ring displays an envelope conformation, with the flap protonated N atom lying 0.497 (3) Å above the mean plane of the remaining four atoms. In the crystal structure, one-dimensional supra­molecular chains parallel to the crystallographic 2_1_ screw axis are formed by N—H⋯Cl hydrogen bonds involving the NH_2_^+^ and Cl^−^ groups. Intra­molecular N—H⋯Cl inter­actions are also present.

## Related literature

For chiral secondary amine catalysts based on the imidazolidinone architecture, see: Ouellet *et al.* (2007 ▶). For Michael additions of aldehydes to enones with a MacMillan imidazol­idinone catalyst, see: Hechavarria Fonseca & List (2004 ▶).
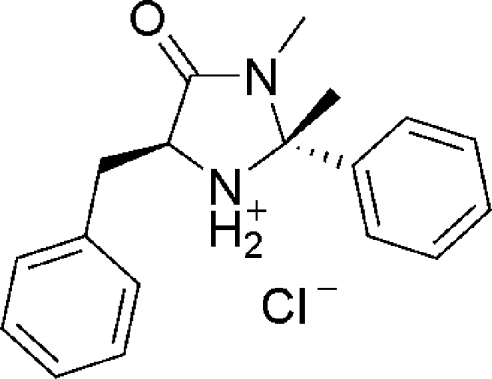


## Experimental

### 

#### Crystal data

C_18_H_21_N_2_O^+^·Cl^−^
*M* *_r_* = 316.83
Monoclinic, 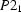

*a* = 10.5797 (11) Å
*b* = 7.5876 (7) Å
*c* = 10.8741 (12) Å
β = 105.516 (3)°
*V* = 841.10 (15) Å^3^
*Z* = 2
Mo *K*α radiation
μ = 0.23 mm^−1^
*T* = 296 K
0.34 × 0.26 × 0.11 mm

#### Data collection

Rigaku R-AXIS RAPID diffractometer
Absorption correction: multi-scan (*ABSCOR*; Higashi, 1995 ▶) *T*_min_ = 0.920, *T*_max_ = 0.975
8064 measured reflections
3371 independent reflections
2933 reflections with *F* ^2^ > 2σ(*F* ^2^)
*R*_int_ = 0.024

#### Refinement

*R*[*F* ^2^ > 2σ(*F* ^2^)] = 0.030
*wR*(*F* ^2^) = 0.073
*S* = 1.00
3371 reflections
201 parameters
H-atom parameters constrained
Δρ_max_ = 0.32 e Å^−3^
Δρ_min_ = −0.30 e Å^−3^
Absolute structure: Flack (1983 ▶), 1323 Friedel pairs
Flack parameter: 0.03 (4)

### 

Data collection: *PROCESS-AUTO* (Rigaku, 1998 ▶); cell refinement: *PROCESS-AUTO*; data reduction: *CrystalStructure* (Rigaku/MSC, 2004 ▶); program(s) used to solve structure: *SIR97* (Altomare *et al.*, 1999 ▶); program(s) used to refine structure: *CRYSTALS* (Betteridge *et al.*, 2003 ▶); molecular graphics: *ORTEPIII* (Burnett & Johnson, 1996 ▶); software used to prepare material for publication: *CRYSTALS*.

## Supplementary Material

Crystal structure: contains datablocks global, I. DOI: 10.1107/S1600536809010460/bh2221sup1.cif

Structure factors: contains datablocks I. DOI: 10.1107/S1600536809010460/bh2221Isup2.hkl

Additional supplementary materials:  crystallographic information; 3D view; checkCIF report

## Figures and Tables

**Table 1:** Hydrogen-bond geometry (Å, °)

*D*—H⋯*A*	*D*—H	H⋯*A*	*D*⋯*A*	*D*—H⋯*A*
N2—H201⋯Cl1	0.86	2.30	3.1170 (14)	160
N2—H202⋯Cl1^i^	0.86	2.24	3.0999 (14)	174

Symmetry code: (i) 
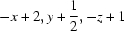
.

**Fig. 1.**
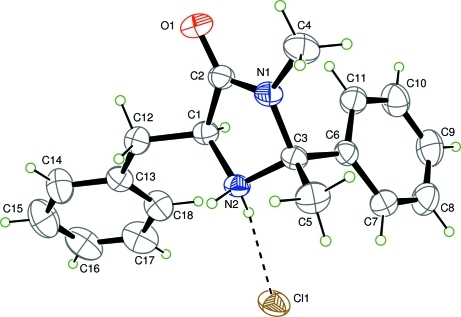
The asymmetric unit of the title compound, with the atomic labelling scheme. Displacement ellipsoids are drawn at the 40% probability level.

**Fig. 2.**
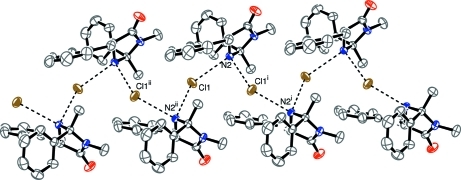
A part of the crystal structure of the title salt, with hydrogen bonds represented with dashed lines.

## supplementary crystallographic information

### Comment

Ten years ago, MacMillan and his laboratory developed chiral secondary amine catalysts based on the imidazolidinone architecture, which has led to the development of over 30 different enantioselective transformations for asymmetric synthesis (Ouellet *et al.*, 2007). In recent years, Michael additions of aldehydes to enones with a MacMillan imidazolidinone catalyst have been reported (Hechavarria Fonseca & List, 2004). The title compound, prepared as a kind of organocatalyst for use in the asymmetric Michael addition of aldehydes to enones, was synthesized from *L*-phenylalanine. The crystal structure and absolute configuration of the title compound are reported in this article.

The compound consists of an ionic pair, a protonated ammonium cation and a Cl^-^ anion (Fig. 1). The chiral atom C1 has the expected *S* configuration, while the other chiral atom C3 was determined to be in a *R* configuration. The C1/C2/C3/N1 atoms of the imidazolidinone ring are almost coplanar. The distance of atom N2 to the C1/C2/C3/N1 mean plane is 0.497 (3) Å, while the distance of atom C12 of the benzyl group to the plane is 0.920 (4) Å. In the crystal structure of the title salt, one-dimensional supramolecular chains are formed, by intra- and inter-molecular N—H···Cl hydrogen bonds (Fig. 2).

### Experimental

To a stirred solution of *L*-phenylalanine (3.87 g, 24 mmol) in dry methanol (50 ml) was added thionyl chloride (2.72 ml, 37.5 mmol). After refluxed for 48 h., the solution was concentrated under vacuum and *L*-phenylalanine methyl ester dichloride crystallized from methanol to give white crystals. To an ethanolic MeNH_2_ solution (8 *M*, 6 ml) was added *L*-phenylalanine methyl ester dihydrochloride (2.41 g, 10 mmol). The solution was stirred at room temperature for 24 h, and then the solvent removed under reduced pressure. To this residue was added MeOH (80 ml), acetophenone (3 g, 25 mmol), and a catalytic amount of *p*-toluenesulfonic acid (30 mg, 0.16 mmol). The resulting solution was refluxed for 24 h and further stirred at room temperature for 2 h. The mixture was subsequently concentrated under reduced pressure, giving the crude product. The resolution of the racemic compounds was by means of column chromatography with methyl and petroleum ether (1:4). The residue was taken up in ethylether and a solution of HCl-dioxane (4.0 *M*) was added to the precipitate. Suitable crystals were obtained by slow evaporation of a methanol solution of the crude at room temperature.

### Refinement

All H atoms were placed in calculated positions, with C—H = 0.93 (aromatic CH), 0.96 (methyl CH_3_), 0.97 (methylene CH_2_) or 0.98 Å (methine CH), and N—H = 0.86 Å. Displacement parameters for H atoms were calculated as *U*_iso_(H) = 1.2*U*_eq_(carrier atom).

### Crystal data

**Table:**

C_18_H_21_N_2_O^+^·Cl^−^	*F*(000) = 336.00
*M**_r_* = 316.83	*D*_x_ = 1.251 Mg m^−^^3^
Monoclinic, *P*2_1_	Mo *K*α radiation, λ = 0.71075 Å
Hall symbol: P 2yb	Cell parameters from 5642 reflections
*a* = 10.5797 (11) Å	θ = 3.1–27.4°
*b* = 7.5876 (7) Å	µ = 0.23 mm^−^^1^
*c* = 10.8741 (12) Å	*T* = 296 K
β = 105.516 (3)°	Needle, colourless
*V* = 841.10 (15) Å^3^	0.34 × 0.26 × 0.11 mm
*Z* = 2	

### Data collection

**Table:**

Rigaku R-AXIS RAPID diffractometer	2933 reflections with *F*^2^ > 2σ(*F*^2^)
Detector resolution: 10.00 pixels mm^-1^	*R*_int_ = 0.024
ω scans	θ_max_ = 27.4°
Absorption correction: multi-scan (*ABSCOR*; Higashi, 1995)	*h* = −13→13
*T*_min_ = 0.920, *T*_max_ = 0.975	*k* = −8→9
8064 measured reflections	*l* = −14→14
3371 independent reflections	

### Refinement

**Table:**

Refinement on *F*^2^	(Δ/σ)_max_ < 0.001
*R*[*F*^2^ > 2σ(*F*^2^)] = 0.030	Δρ_max_ = 0.32 e Å^−^^3^
*wR*(*F*^2^) = 0.073	Δρ_min_ = −0.30 e Å^−^^3^
*S* = 1.00	Extinction correction: *CRYSTALS* (Betteridge et al., 2003)
3371 reflections	Extinction coefficient: 148 (16)
201 parameters	Absolute structure: Flack (1983), 1323 Friedel pairs
H-atom parameters constrained	Flack parameter: 0.03 (4)
*w* = 1/[0.0003*F*_o_^2^ + 1.04σ(*F*_o_^2^)]/(4*F*_o_^2^)	

### Fractional atomic coordinates and isotropic or equivalent isotropic displacement parameters (Å^2^)

**Table:**

	*x*	*y*	*z*	*U*_iso_*/*U*_eq_	
Cl1	0.93865 (4)	0.37096 (8)	0.38333 (4)	0.04566 (11)	
O1	0.55546 (12)	0.94486 (19)	0.53994 (12)	0.0482 (3)	
N1	0.66144 (12)	0.93555 (17)	0.38197 (12)	0.0328 (3)	
N2	0.80291 (11)	0.70805 (17)	0.45120 (12)	0.0282 (3)	
C1	0.70374 (14)	0.7044 (2)	0.52767 (14)	0.0300 (4)	
C2	0.63026 (12)	0.8761 (2)	0.48651 (13)	0.0329 (3)	
C3	0.73966 (14)	0.8101 (2)	0.33039 (14)	0.0299 (3)	
C4	0.6042 (2)	1.0933 (2)	0.3114 (2)	0.0506 (5)	
C5	0.84477 (16)	0.9014 (2)	0.28214 (17)	0.0433 (5)	
C6	0.65243 (14)	0.6841 (2)	0.23338 (14)	0.0308 (4)	
C7	0.70586 (18)	0.5811 (2)	0.15399 (17)	0.0424 (5)	
C8	0.6288 (2)	0.4612 (2)	0.07068 (18)	0.0539 (6)	
C9	0.4987 (2)	0.4414 (2)	0.06476 (19)	0.0538 (5)	
C10	0.44421 (19)	0.5420 (2)	0.14153 (19)	0.0506 (5)	
C11	0.51948 (16)	0.6636 (2)	0.22594 (16)	0.0395 (4)	
C12	0.76199 (17)	0.6939 (2)	0.67155 (14)	0.0353 (4)	
C13	0.80340 (14)	0.5137 (2)	0.72774 (17)	0.0347 (4)	
C14	0.8565 (2)	0.4999 (2)	0.8593 (2)	0.0530 (5)	
C15	0.8969 (2)	0.3397 (3)	0.9158 (2)	0.0684 (7)	
C16	0.8867 (2)	0.1899 (3)	0.8436 (2)	0.0618 (6)	
C17	0.8346 (2)	0.2009 (2)	0.7136 (2)	0.0582 (6)	
C18	0.79285 (17)	0.3619 (2)	0.65706 (18)	0.0467 (4)	
H1	0.6438	0.6050	0.4996	0.036*	
H7	0.7941	0.5929	0.1569	0.051*	
H8	0.6658	0.3932	0.0182	0.065*	
H9	0.4478	0.3602	0.0089	0.065*	
H10	0.3557	0.5289	0.1373	0.061*	
H11	0.4811	0.7314	0.2775	0.047*	
H14	0.8647	0.6005	0.9097	0.064*	
H15	0.9314	0.3332	1.0038	0.082*	
H16	0.9148	0.0821	0.8819	0.074*	
H17	0.8274	0.1001	0.6636	0.070*	
H18	0.7568	0.3672	0.5692	0.056*	
H41	0.5471	1.1496	0.3547	0.061*	
H42	0.5549	1.0606	0.2269	0.061*	
H43	0.6729	1.1732	0.3063	0.061*	
H51	0.8041	0.9742	0.2103	0.052*	
H52	0.8988	0.8145	0.2568	0.052*	
H53	0.8980	0.9732	0.3489	0.052*	
H121	0.6969	0.7390	0.7115	0.042*	
H122	0.8388	0.7692	0.6932	0.042*	
H201	0.8213	0.6026	0.4327	0.034*	
H202	0.8734	0.7600	0.4934	0.034*	

### Atomic displacement parameters (Å^2^)

**Table:**

	*U*^11^	*U*^22^	*U*^33^	*U*^12^	*U*^13^	*U*^23^
Cl1	0.0397 (2)	0.0372 (2)	0.0553 (2)	0.0126 (2)	0.00437 (17)	−0.0024 (2)
O1	0.0443 (6)	0.0550 (8)	0.0494 (7)	0.0169 (5)	0.0195 (5)	−0.0050 (5)
N1	0.0349 (6)	0.0262 (6)	0.0368 (7)	0.0080 (5)	0.0084 (5)	0.0012 (5)
N2	0.0240 (5)	0.0249 (7)	0.0344 (6)	0.0015 (5)	0.0056 (5)	−0.0035 (5)
C1	0.0271 (7)	0.0279 (8)	0.0354 (8)	−0.0013 (6)	0.0091 (6)	−0.0020 (6)
C2	0.0269 (6)	0.0344 (8)	0.0357 (8)	0.0021 (8)	0.0056 (5)	−0.0057 (8)
C3	0.0289 (7)	0.0261 (8)	0.0340 (8)	0.0033 (6)	0.0074 (6)	0.0013 (6)
C4	0.0570 (11)	0.0356 (10)	0.0565 (12)	0.0167 (9)	0.0107 (9)	0.0075 (8)
C5	0.0408 (8)	0.0405 (11)	0.0521 (10)	−0.0058 (8)	0.0188 (8)	0.0026 (8)
C6	0.0337 (7)	0.0297 (8)	0.0276 (7)	0.0034 (7)	0.0058 (6)	0.0025 (6)
C7	0.0430 (9)	0.0445 (10)	0.0392 (9)	0.0087 (8)	0.0100 (8)	−0.0045 (8)
C8	0.0678 (12)	0.0526 (12)	0.0392 (10)	0.0090 (11)	0.0109 (9)	−0.0113 (9)
C9	0.0638 (12)	0.0493 (11)	0.0408 (10)	−0.0109 (10)	0.0009 (9)	−0.0103 (8)
C10	0.0413 (9)	0.0615 (13)	0.0448 (11)	−0.0121 (9)	0.0042 (8)	−0.0023 (9)
C11	0.0337 (8)	0.0477 (11)	0.0376 (9)	−0.0032 (8)	0.0103 (7)	−0.0031 (8)
C12	0.0388 (8)	0.0333 (9)	0.0342 (8)	−0.0027 (7)	0.0104 (7)	−0.0009 (7)
C13	0.0311 (8)	0.0350 (9)	0.0384 (9)	−0.0029 (7)	0.0100 (7)	0.0036 (7)
C14	0.0629 (12)	0.0495 (12)	0.0408 (10)	−0.0015 (10)	0.0037 (9)	0.0017 (9)
C15	0.0731 (13)	0.0708 (19)	0.0514 (12)	0.0022 (13)	−0.0005 (10)	0.0230 (12)
C16	0.0491 (11)	0.0464 (13)	0.0818 (17)	−0.0030 (10)	0.0035 (11)	0.0232 (12)
C17	0.0566 (11)	0.0342 (11)	0.0807 (15)	−0.0043 (9)	0.0129 (11)	0.0047 (10)
C18	0.0484 (9)	0.0387 (9)	0.0496 (10)	−0.0024 (10)	0.0072 (7)	0.0003 (10)

### Geometric parameters (Å, °)

**Table:**

O1—C2	1.218 (2)	C17—C18	1.385 (2)
N1—C2	1.344 (2)	N2—H201	0.860
N1—C3	1.467 (2)	N2—H202	0.860
N1—C4	1.463 (2)	C1—H1	0.980
N2—C1	1.503 (2)	C4—H41	0.960
N2—C3	1.5176 (19)	C4—H42	0.960
C1—C2	1.521 (2)	C4—H43	0.960
C1—C12	1.523 (2)	C5—H51	0.960
C3—C5	1.518 (2)	C5—H52	0.960
C3—C6	1.536 (2)	C5—H53	0.960
C6—C7	1.392 (2)	C7—H7	0.930
C6—C11	1.396 (2)	C8—H8	0.930
C7—C8	1.385 (2)	C9—H9	0.930
C8—C9	1.368 (3)	C10—H10	0.930
C9—C10	1.367 (3)	C11—H11	0.930
C10—C11	1.392 (2)	C12—H121	0.970
C12—C13	1.514 (2)	C12—H122	0.970
C13—C14	1.394 (2)	C14—H14	0.930
C13—C18	1.373 (2)	C15—H15	0.930
C14—C15	1.378 (3)	C16—H16	0.930
C15—C16	1.369 (3)	C17—H17	0.930
C16—C17	1.375 (3)	C18—H18	0.930
			
C2—N1—C3	113.37 (13)	C2—C1—H1	109.3
C2—N1—C4	123.90 (16)	C12—C1—H1	109.3
C3—N1—C4	121.80 (15)	N1—C4—H41	109.5
C1—N2—C3	106.07 (11)	N1—C4—H42	109.5
N2—C1—C2	101.48 (12)	N1—C4—H43	109.5
N2—C1—C12	114.76 (12)	H41—C4—H42	109.5
C2—C1—C12	112.44 (13)	H41—C4—H43	109.5
O1—C2—N1	126.71 (16)	H42—C4—H43	109.5
O1—C2—C1	124.95 (15)	C3—C5—H51	109.5
N1—C2—C1	108.34 (14)	C3—C5—H52	109.5
N1—C3—N2	99.40 (12)	C3—C5—H53	109.5
N1—C3—C5	112.12 (13)	H51—C5—H52	109.5
N1—C3—C6	111.70 (12)	H51—C5—H53	109.5
N2—C3—C5	109.75 (11)	H52—C5—H53	109.5
N2—C3—C6	108.76 (12)	C6—C7—H7	119.7
C5—C3—C6	114.07 (14)	C8—C7—H7	119.7
C3—C6—C7	120.44 (14)	C7—C8—H8	119.6
C3—C6—C11	121.35 (15)	C9—C8—H8	119.6
C7—C6—C11	118.15 (14)	C8—C9—H9	120.2
C6—C7—C8	120.59 (18)	C10—C9—H9	120.2
C7—C8—C9	120.8 (2)	C9—C10—H10	119.6
C8—C9—C10	119.57 (18)	C11—C10—H10	119.6
C9—C10—C11	120.81 (18)	C6—C11—H11	119.9
C6—C11—C10	120.13 (17)	C10—C11—H11	119.9
C1—C12—C13	117.13 (14)	C1—C12—H121	107.5
C12—C13—C14	118.38 (16)	C1—C12—H122	107.5
C12—C13—C18	124.10 (15)	C13—C12—H121	107.5
C14—C13—C18	117.52 (17)	C13—C12—H122	107.5
C13—C14—C15	121.02 (19)	H121—C12—H122	109.5
C14—C15—C16	120.6 (2)	C13—C14—H14	119.5
C15—C16—C17	119.2 (2)	C15—C14—H14	119.5
C16—C17—C18	120.2 (2)	C14—C15—H15	119.7
C13—C18—C17	121.50 (17)	C16—C15—H15	119.7
C1—N2—H201	110.3	C15—C16—H16	120.4
C1—N2—H202	110.3	C17—C16—H16	120.4
C3—N2—H201	110.3	C16—C17—H17	119.9
C3—N2—H202	110.3	C18—C17—H17	119.9
H201—N2—H202	109.5	C13—C18—H18	119.2
N2—C1—H1	109.3	C17—C18—H18	119.2
			
C2—N1—C3—N2	−25.51 (14)	N2—C3—C6—C7	−85.03 (19)
C2—N1—C3—C5	−141.42 (12)	N2—C3—C6—C11	92.14 (17)
C2—N1—C3—C6	89.12 (15)	C5—C3—C6—C7	37.8 (2)
C3—N1—C2—O1	−171.77 (14)	C5—C3—C6—C11	−145.00 (16)
C3—N1—C2—C1	7.66 (16)	C3—C6—C7—C8	176.89 (16)
C4—N1—C2—O1	−2.7 (2)	C3—C6—C11—C10	−176.77 (16)
C4—N1—C2—C1	176.74 (13)	C7—C6—C11—C10	0.5 (2)
C4—N1—C3—N2	165.15 (13)	C11—C6—C7—C8	−0.4 (2)
C4—N1—C3—C5	49.24 (18)	C6—C7—C8—C9	−0.0 (2)
C4—N1—C3—C6	−80.22 (18)	C7—C8—C9—C10	0.3 (3)
C1—N2—C3—N1	33.21 (13)	C8—C9—C10—C11	−0.2 (3)
C1—N2—C3—C5	150.92 (13)	C9—C10—C11—C6	−0.2 (2)
C1—N2—C3—C6	−83.66 (14)	C1—C12—C13—C14	−179.68 (17)
C3—N2—C1—C2	−29.59 (13)	C1—C12—C13—C18	0.8 (2)
C3—N2—C1—C12	−151.10 (12)	C12—C13—C14—C15	−179.5 (2)
N2—C1—C2—O1	−166.51 (14)	C12—C13—C18—C17	178.83 (18)
N2—C1—C2—N1	14.06 (14)	C14—C13—C18—C17	−0.7 (2)
N2—C1—C12—C13	−81.41 (18)	C18—C13—C14—C15	0.1 (2)
C2—C1—C12—C13	163.27 (14)	C13—C14—C15—C16	0.5 (3)
C12—C1—C2—O1	−43.4 (2)	C14—C15—C16—C17	−0.5 (3)
C12—C1—C2—N1	137.17 (14)	C15—C16—C17—C18	−0.1 (2)
N1—C3—C6—C7	166.27 (15)	C16—C17—C18—C13	0.7 (3)
N1—C3—C6—C11	−16.6 (2)		

### Hydrogen-bond geometry (Å, °)

**Table:**

*D*—H···*A*	*D*—H	H···*A*	*D*···*A*	*D*—H···*A*
N2—H201···Cl1	0.86	2.30	3.1170 (14)	160
N2—H202···Cl1^i^	0.86	2.24	3.0999 (14)	174

Symmetry codes: (i) −*x*+2, *y*+1/2, −*z*+1.

## References

[bb1] Altomare, A., Burla, M. C., Camalli, M., Cascarano, G. L., Giacovazzo, C., Guagliardi, A., Moliterni, A. G. G., Polidori, G. & Spagna, R. (1999). *J. Appl. Cryst.* **32**, 115–119.

[bb2] Betteridge, P. W., Carruthers, J. R., Cooper, R. I., Prout, K. & Watkin, D. J. (2003). *J. Appl. Cryst.* **36**, 1487.

[bb3] Burnett, M. N. & Johnson, C. K. (1996). *ORTEPIII* Report ORNL-6895. Oak Ridge National Laboratory, Tennessee, USA.

[bb4] Flack, H. D. (1983). *Acta Cryst.* A**39**, 876–881.

[bb5] Hechavarria Fonseca, M. T. & List, B. (2004). *Angew. Chem. Int. Ed* **43**, 3958–3960. 10.1002/anie.200460578 15274225

[bb6] Higashi, T. (1995). *ABSCOR* Rigaku Corporation, Tokyo, Japan.

[bb7] Ouellet, S. G., Walji, A. B. & Macmillan, D. W. C. (2007). *Acc. Chem. Res* **40**, 1327–1339. 10.1021/ar7001864 18085748

[bb8] Rigaku (1998). *PROCESS-AUTO* Rigaku Corporation, Tokyo, Japan.

[bb9] Rigaku/MSC (2004). *CrystalStructure* Rigaku/MSC, The Woodlands, Texas, USA.

